# HDAPD: a web tool for searching the disease-associated protein structures

**DOI:** 10.1186/1471-2105-11-88

**Published:** 2010-02-17

**Authors:** Yi-Ruen Lin, Hsin-Yuan Wei, Tsung-Lin Tsai, Thy-Hou Lin

**Affiliations:** 1 Institute of Molecular Medicine and Department of Life Science, National Tsing Hua University, HsinChu, 30013, Taiwan, Republic of China

## Abstract

**Background:**

The protein structures of the disease-associated proteins are important for proceeding with the structure-based drug design to against a particular disease. Up until now, proteins structures are usually searched through a PDB id or some sequence information. However, in the HDAPD database presented here the protein structure of a disease-associated protein can be directly searched through the associated disease name keyed in.

**Description:**

The search in HDAPD can be easily initiated by keying some key words of a disease, protein name, protein type, or PDB id. The protein sequence can be presented in FASTA format and directly copied for a BLAST search. HDAPD is also interfaced with Jmol so that users can observe and operate a protein structure with Jmol. The gene ontological data such as cellular components, molecular functions, and biological processes are provided once a hyperlink to Gene Ontology (GO) is clicked. Further, HDAPD provides a link to the KEGG map such that where the protein is placed and its relationship with other proteins in a metabolic pathway can be found from the map. The latest literatures namely titles, journals, authors, and abstracts searched from PubMed for the protein are also presented as a length controllable list.

**Conclusions:**

Since the HDAPD data content can be routinely updated through a PHP-MySQL web page built, the new database presented is useful for searching the structures for some disease-associated proteins that may play important roles in the disease developing process for performing the structure-based drug design to against the diseases.

## Background

Most of the current disease databases designed can provide clinical information such as features, syndromes, diagnosis methods and therapy for doctors, pharmacists, nurses and medical technology staffs who work in the clinic settings. The disease-associated databases remain fewer as compared with the molecular biological databases built so far. The Online Mendelian Inheritance in Man (OMIM) [1] of NCBI and GeneCards [2] are the two disease databases built that can provide relationship between diseases and genes. Genes and Disease [3] is a database built for collecting articles and discussing genes that derive the diseases. The International Classification of Diseases (ICD) [4] is a WHO database that supplies diagnostic classification for diseases, analyzes general health situation for population groups, and monitors incidence and prevalence of diseases and other health related problems.

The disease-related protein structures are of great research interest for both experimental and computational scientists. Their importance stems from the fact that they provide molecular pictures of disease processes, a necessary prerequisite for structure-based drug design. In this work, we have developed a web searching tool Human Disease-Associated Protein Database (HDAPD) for searching the disease-associated protein structures for interested researchers. The web searching tool can be initiated by typing some related keywords for searching the information on structures, structure-related sequences, location, function, pathway, and literatures for a disease-associated protein collected in the database.

## Construction and content

The sources of data used in HDAPD include two parts namely databases and literatures. The disease-associated proteins were mined from the research articles, and databases Genes and Disease, OMIM, and ICD. The ICD classification for diseases was followed for grouping proteins collected for each disease. A background introduction extracted from database Genetics Home Reference (GHR) [5] or online publication of GeneReviews [6] was listed for each disease name clicked. The overall disease and literature information was extracted from databases OMIM, Genes and Disease, ICD, PubMed [7], GeneReviews, and GHR while the associated protein information such as name, structure, sequence, classification, PDB id, ontological data, and KEGG map was extracted from databases PDB [8], UniProt [9], GO [10], and KEGG [11], respectively. We searched the proteins that were associated with the diseases from database Genes and Diseases first and then fed this information into database OMIM for mining more potential proteins. These disease-associated proteins were grouped together based on the disease caused by them. We also extracted the disease information from database ICD and these were similarly processed as those extracted from database Genes and Diseases. The gathered information for diseases and disease-related proteins were classified and tabulated using the MySQL [12] format. Meanwhile, the background information of each disease collected was extracted from databases GeneReviews and GHR and recorded as text files. The disease name was treated as header or footer for separating each disease introduction and searching the correct paragraph where the disease-related information was recorded. The contents of diseases plus a brief introduction for each disease and the total disease-associated proteins collected in HDAPD were presented as pull-down lists once the corresponding hyperlinks given in the front page were clicked.

## Utility

The Perl scripts [13] were used for dividing a group of proteins and sending these proteins to database UniprotKB for searching the corresponding PDB codes. More comprehensive information such as protein description, donation organism, classification, experimental type, and X-ray structure corresponding to each protein searched was extracted through the wiped FTP archive. A unique table in MySQL format was generated for each protein searched with a PDB entry. We also integrated a molecular viewer in HDAPD so that users can view the structures of proteins collected in the database. A hyperlink to Jmol [14], a molecular viewer written with Java Applet [15], was built in HDAPD for users to directly viewing and operating the structure of a protein searched. The functional bar in Jmol could be initiated once the cursor was moved into the Jmol template and the mouse's right button was clicked. In addition to using the functional bar, the usage of a Jmol script could also be viewed and used from the "Jmol interactive scripting documentation" via a hyperlink clicked. The desired Jmol script could be entered into a box provided below the Jmol template for operating the protein molecule.

The protein sequences were collected from the wiped FTP archives and saved as a huge text file. This text file was separated in alphabetic order according to the second word identified from a PDB code. The protein sequence of a protein could be assessed from the corresponding protein page presented by clicking the button ProteinSequence on the bottom. The PDB id, molecular type, length, and name were presented ahead of sequence. The protein sequence was also presented in FASTA [16] format and could be directly copied into BLAST [17] for other searching tasks if the hyperlink "FASTA format" located on upper right of the page was clicked.

The GO Consortium rules use a consistent language and controlled vocabularies to describing the role of genes and related proteins including their cellular component, molecular function, and biological process for eukaryotic organisms. The protein structures in GO Human are collected from UniProt while those in GO Rattus Norvegicus are collected from the Rat Genome Database [18]. To assess the GO data, a protein name typed in HDAPD was converted to an internal id for identifying the GO ids. The GO ids were consisted of three parts according to the three ontological data such as location, function, and description provided by the database. These were presented in three tables with each being divided into two parts, the left and right portions. While the GO ids were recorded in the left portion, the corresponding definitions were given in the right one. The GO ids can be keyed into AmiGO [19], a web tool designed by GO, for obtaining more useful data such as term linkages from the GO database. We collected a variety of protein names and ids from several databases and kept this information as tables in HDAPD. The KEGG protein and pathway ids acquired from Uniprot were used to assess the KEGG maps which would provide useful information such as the role of a protein in a metabolic pathway and whether or not the protein would interact with other proteins in the same pathway. When the KEGG button of a hyperlink built for each collected protein in HDAPD was clicked, a table containing two portions with left one gives the hyperlinks of pathways while right one gives the names of the corresponding pathways was presented. Clicking on the hyperlink of a pathway would link HDAPD to the KEGG server and bring up the corresponding KEGG analyses and graphic illustrations. HDAPD also provides a hyperlink to database PubMed of NCBI in the protein page. This was initiated by a keyword generated through a Perl script and sent to the NCBI server. The information extracted by HDAPD from PubMed was titles, journals, authors, and abstracts published for an interested protein structure searched and these were presented as a long list. Not only the length of this list is controllable, the content of this list could be also sorted in order of author, last author, journal, or publication date.

## Discussion

**Figure 1:**
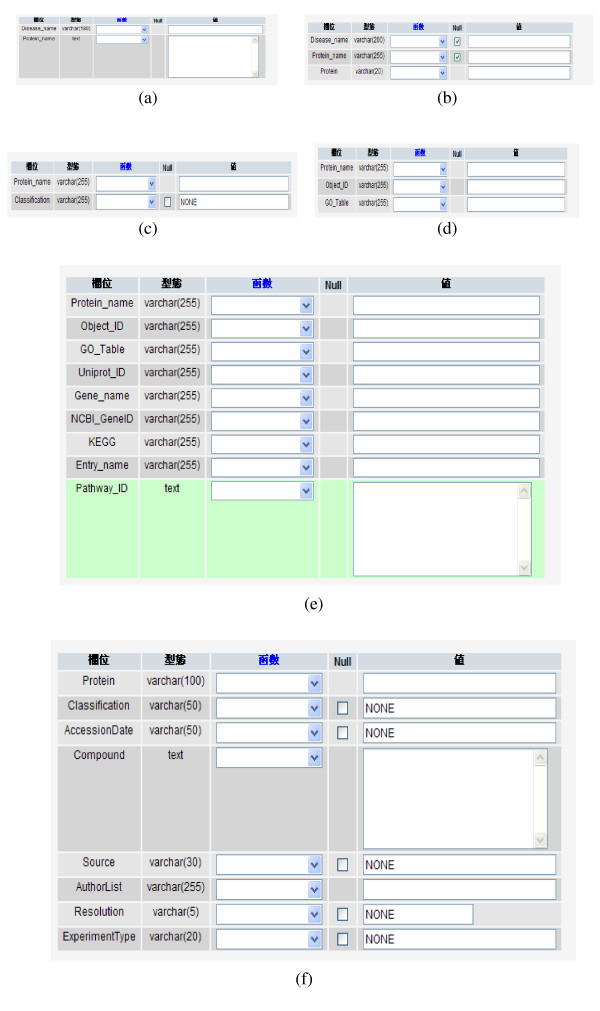
**The protein structures collected in HDAPD can be routinely updated through six PHP-MySQL templates designed namely (a), (b), (c), (d), (e), and (f)**. These templates are used for entering (a) disease and protein names; (b) disease and protein classification; (c) disease and PDB code; (d) protein names, UniProt and GO id; (e) protein name, UniProt id, GO id, gene name, NCBI id, and KEGG id; and (f) protein name, protein description, source, authors, resolution, and method.

The protein structures collected in HDAPD can be routinely updated through six PHP-MySQL templates designed as shown in Figure 1. A comparison for database contents and searching functions provided by HDAPD with those provided by databases NCBI Entrez, EMBL, UniProt, and GHR is shown in Table 1. Apparently, while the other databases providing more information on protein sequence, gene ontology, and protein structure, only disease-related information including literatures are supplied by database GHR (Table 1). Moreover, there are no disease list, disease introduction for each disease, and disease-associated protein lists provided by both NCBI Entrez and EMBL databases. The UniProt database does give an introduction for each disease but provide no list in diseases and diseases-associated proteins (Table 1). Undoubtedly, NCBI Entrez, EMBL, and UniProt are far more superior to HDAPD in providing information on protein sequences either determined or annotated (Table 1). In fact, most of the protein sequences in HDAPD are collected from PDB and are therefore associated with the corresponding structures determined. By typing in some keywords, a protein list with PDB id, classification, and taxonomy information attached for each protein are provided by NCBI Entrez, EMBL, and UniProt. However, HDAPD takes only keywords in diseases or disease-associated proteins for searching protein structures. The protein lists provided by HDAPD are also classified based on their functions and then hyperlinks for PDB, GO, KEGG, and PubMed are provided if a protein on a list is clicked. Note that no GO information is provided by NCBI Entrez. However, the KEGG pathway description and maps are provided by all the databases compared except GHR (Table 1). Moreover, all the databases compared except GHR are able to provide literature searching results for the protein structures searched. However, the literatures searched in HDAPD can be selected and sorted in alphabetic order of first author, last author, and journal name, or in chronologic order of publication date.

**Table 1:** A comparison for database contents and searching functions provided by HDAPD with those provided by databases NCBI Entrez, EMBL, UniProt, and GHR

	Databases	HDAPD	NCBI Entrez	EMBL	UniProt	GHR
	
	Web site	http://140.114.100.145/index.html	http://www.ncbi.nlm.nih.gov/sites/gquery	http://www.ebi.ac.uk/embl/	http://www.uniprot.org/	http://ghr.nlm.nih.gov/
Functions	Disease types	ICD-10; Genes and Disease (classified diseases into 14 groups; 285 diseases)				(classified diseases into 17 groups)
	
	Disease list	∘				∘

Disease introduction		Gene Review; Genetics Home Reference				
	
	Disease introduction	∘			∘	∘

Disease-associated proteins		ICD-10; Genes and Disease; OMIM				
	
	Disease-associated protein list	∘	*	*	*	
				
			*A protein list is provided by typing in keywords.			

Determine-d and annotated protein sequences			SwissProt, PIR, PRF, PDB,	PRIDE UniProtKB UniRef UniParc	UniProtKB UniRef UniParc	
	
	Sequence database		∘	∘	∘	

Determine-d protein structure	3-D macromolecular structures	PDB (3,189 all are diseases-associated)	MMDB (over 20000 structures, but not disease-associated)	PDBe	PDB PDBe	
	
	PDB ID	∘	∘	∘	∘	
	
	Compound	∘	∘	∘	◎	
	
	Classification	∘	∘	∘	◎	
	
	Source	∘	◎	∘	◎	
	
	Resolution	∘	◎	∘	∘	
	
	Method	∘	◎	∘	∘	
	
	Author List	∘	∘	∘	◎	
	
	Accession Date	∘	∘	∘	◎	
	
	Protein sequence (Primary)	∘	∘	∘	◎	
	
	Molecular viewer	∘	◎	∘	◎	
	
	◎: A hyperlink to PDB is provided for a search result.					

Gene ontology		GO	Taxonomy	BioCatalogue GO SBO Taxonomy	GO Taxonomy	
	
	Gene Ontology (GO)	∘	◎	∘	∘	
			
		◎: A hyperlink to PubMed of NCBI is provided for a search result.				

Pathway	Pathway and systems of interacting molecules	KEGG	KEGG Reactome	BioModels Reactome Rhea	UniProtKB/Swiss-Prot	
	
	Pathway description	∘	∘	∘	∘	
	
	Pathway map	∘	∘	∘	∘	

Literature	Full text and journal articles	PubMed	PubMed	Medline Patents	PubMed SRS CiteXplore	PubMed
	Literature extracting	∘	∘	∘	∘	
	
	Author	∘	∘	∘	∘	◎
	
	Journal	∘	∘	∘	∘	◎
	
	Relative date	∘	∘	∘	∘	◎
	
	Sorting	∘				
	
	Date	∘	∘	∘	∘	
	
	◎: A hyperlink to PubMed of NCBI is provided for a search result.					

PRIDE: *Proteomics identification database*, UniProtKB: *UniProt knowledge base of protein sequences*, UniRef: *UniProt Non-redundant reference databases*, UniParc: *Non-redundant archive of protein sequences*, PDB: *Protein database bank*, MMDB: *The Molecular modeling database*, PDBe: *Macromolecular structures database*, GO: *Gene ontology*, Taxonomy: *NCBI Taxonomy database of organism names*, BioCatalogue: *BioCatalogue*, SBO: *Systems biology ontology*, KEGG: *Kyoto encyclopedia of genes and genomes*, Reactome: *Database of core biochemical pathways and reactions*, BioModels: *Database of mathematical models of biological interest*, Rhea: *Manually annotated database of chemical reactions created in collaboration with the Swiss Institute of Bioinformatics (SIB)*, PubMed: *PubMed of NCBI*, Medline: *Citations and abstracts from many life-science journals*, Patents: *Biology-related abstracts of patent applications*.

The searched results for two diseases and a disease-associated protein namely Lung cancer, Diabetes, and Tumor protein 53 by HDAPD and by all the four databases compared are shown in Table 2. This table contrasts the major difference between HDAPD and the other four databases compared in that all the diseases-associated proteins collected in the former are classified into different disease groups while no such classification is given in the latter. Therefore, the structure of a disease-associated protein can be more conveniently searched through HDAPD though more information on sequence, taxonomy, and genome are provided by the other databases (Table 2). Except those searched by UniProt, more structures of Lung cancer-associated proteins and Tumor protein 53 are searched by HDAPD than by NCBI Entrez and EMBL (Table 2). HDAPD also gives more protein structures for Tumor protein 53 than those given by both NCBI Entrez and EMBL (Table 2). However, more structures of Diabetes-associated proteins are searched by UniProt than by HDAPD (Table 2). Except HDAPD and UniProt, the GO-related information is indirectly provided by NCBI Entrez or unavailable in EMBL and GHR (Table 2). In general, UniProt does provide much more GO information than HDAPD since the latter only focused on protein structures while the former on all relevant protein sequences. This is also true between the number of literatures searched by HDAPD and those by both NCBI Entrez and EMBL (Table 2). However, more KEGG pathway information for both Lung cancer and Diabetes is provided by HDAPD than by all the other databases compared (Table 2).

**Table 2:** The searched results for two diseases and a disease-associated protein namely Lung cancer, Diabetes, and Tumor protein 53 by HDAPD and NCBI Entrez, EMBL, UniProt, and GHR are compared.

	describe	HDAPD	NCBI Entrez	EMBL	UniProt	GHR
Disease & protein	Lung cancer	293			699	
	
	tumor protein 53	1			1	
	
	diabetes	161			705	

Protein sequences	Lung cancer	-	20314	1524	765	
	
	tumor protein 53	-	679	35	32	
	
	diabetes	-	19353	751	991	

Protein structure	Lung cancer	2155	17	33	2360	
	
	tumor protein 53	61	1	22	79	
	
	diabetes	906	254	547	1082	

GO: biological process	Lung cancer	1872	*none*	0	625	
	
	tumor protein 53	48	*none*	0	31	
	
	diabetes	1127	*none*	1	902	

GO: cellular component	Lung cancer	2017	*none*	0	668	
	
	tumor protein 53	11	*none*	0	31	
	
	diabetes	1080	*none*	0	895	

GO: molecular function	Lung cancer	1953	*none*	0	662	
	
	tumor protein 53	12	*none*	0	27	
	
	diabetes	1096	*none*	0	862	

Number of KEGG paths involved	Lung cancer	893	56	1	26	
	
	tumor protein 53	20	128	0	2	
	
	diabetes	354	65	21	60	

literature	Lung cancer	651248	173048	105635	2876	
	
	tumor protein 53	3831	15739	2818	251	
	
	diabetes	13635	339343	325435	11155	

* The GO information in **NCBI Entrez **is indirectly provided through a hyperlink to **PDB**.

## Conclusions

In this work, we present a comprehensive web tool HDAPD for searching a variety of important information such as name, sequence, structure, ontological data, metabolic pathway data, and relevant literatures for some disease-associated protein structures collected in the database. Currently, the total number of diseases and disease-associated protein structures collected in the database are 454 and 11657, respectively. Moreover, the number of proteins collected by HDAPD with available GO ontological and KEGG metabolic pathway id's are 1086 and 1079, respectively. These numbers will be gradually increased in the future to reflect the new developments and advancements in the related fields since the HDAPD data content can be routinely updated through a PHP-MySQL web page built. The new database presented is useful for searching the structures for some disease-associated proteins that may play important roles in the disease developing process for performing the structure-based drug design to against the diseases.

## Availability and Requirements

**Project name**: building a web tool for searching some disease-related protein structures

**Project home page**: http://www.life.nthu.edu.tw/~lslth, HDAPD can be freely assessed through the following URL: http://140.114.100.145/index.html

**Operating system**: Windows XP

**Programming language**: Perl, PHP, and MySQL

**License**: None

**Any restriction to use by academics**: None

## Authors' contributions

YRL wrote most of the source codes for HDAPD, while HYW and TLT conducted the testing and THL wrote the manuscript. All the authors read and approved the final manuscript.
